# The memory ameliorating effects of novel *N*-benzyl pyridine-2-one derivatives on scopolamine-induced cognitive deficits in mice

**DOI:** 10.1186/s42826-023-00187-y

**Published:** 2024-03-11

**Authors:** Swati Pant, Mohan Gupta, Tulika Anthwal, Monika Chauhan, Sumitra Nain

**Affiliations:** Department of Pharmacy, Banasthali Vidyapith, Banasthali, Tonk, Rajasthan India

**Keywords:** *N*-Benzyl pyridine-2-one, Alzheimer’s disease, Scopolamine, Donepezil, Acetylcholinesterase

## Abstract

**Background:**

Alzheimer's disease (AD), the most common form of progressive dementia in the elderly, is a chronic neurological disorder that decreases cognitive ability. Although the underlying cause of AD is yet unknown, oxidative stress and brain acetylcholine shortage are the key pathogenic causes.

**Results:**

The current study shows that these derivatives have the potential to improve memory in mice by inhibiting scopolamine-induced acetylcholinesterase activity, oxidative and nitrosative stress, and improving locomotor activity and muscle grip strength in the rota rod test. When compared to the illness control, the memory-enhancing potential of novel *N*-benzyl pyridine-2-one derivatives was highly significant (P < 0.0001).

**Conclusions:**

The observed memory ameliorating effect of novel *N*-benzyl pyridine-2-one makes them as a a good choice for treatment of individuals with cognitive impairment.

**Graphical abstract:**

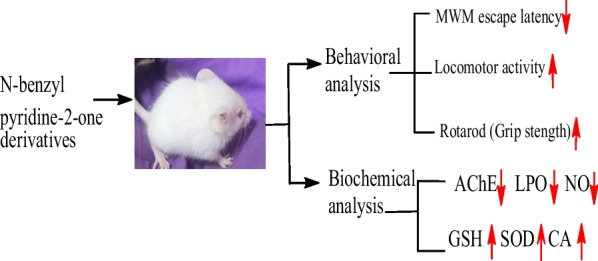

**Supplementary Information:**

The online version contains supplementary material available at 10.1186/s42826-023-00187-y.

## Background

Alzheimer's disease (AD) is a progressive neurological disorder that causes persistent memory and cognitive impairment [1]. According to Alzheimer's Disease International, the number of AD patients will increase from 50 to 152 million by 2050, imposing a significant social and economic burden on patients and their families [2, 3].

Although the precise origin of Alzheimer's disease (AD) is unknown, a variety of factors, including a drop in acetylcholine (ACh) levels, amyloid peptide (A) building, hyperphosphorylated tau-protein deposition, and an increase in oxidative stress, appear to play a role in its start and progression [4]. Studies have shown that acetylcholine levels rise when acetylcholinesterase (AChE) is suppressed, improving cognitive function and memory in AD patients [5]. According to the cholinergic hypothesis, decreasing ACh levels in the brain are the primary cause of cognitive impairment in Alzheimer's disease.

As a result, the most important therapeutic technique is to boost ACh function in the CNS by blocking its enzymatic breakdown by AChE and its related enzyme butyrylcholinesterase (BuChE) [6]. Indeed, tacrine, donepezil, rivastigmine, and galanthamine are among the acetylcholinesterase inhibitors approved by the Food and Drug Administration (FDA) for use in the treatment of Alzheimer's disease. These drugs can benefit persons with cognitive impairment and alleviate disease symptoms [7, 8]. Unfortunately, these AChE inhibitors have had poor clinical outcomes, providing only brief improvements and having little potential to delay the progression of the disease [9, 10]. Finding and creating drugs that can have an AD-modifying effect is thus one of the most difficult challenges that medicinal chemists face.

Heterocyclic compounds are widely used in the pharmaceutical and therapeutic industries because of the variety of their molecular configurations. Pyridine and its derivatives are among those that are attracting a lot of attention because of their broad range of biological possibilities. Medicinal chemists have developed and employed a multitude of pyridine scaffolds to produce novel drugs with a range of pharmacological effects.

The goal of the current investigation was to assess the therapeutic potential of a number of new *N*-benzyl pyridine-2-one derivatives against AD using in-vivo tests. These derivatives were created earlier and assessed using the molecular docking method (Fig. 5) [11]. The molecular docking investigations against AChE enzymes yielded encouraging results. Our prior study's findings led us to look at these novel compounds in more detail using animal models of AD.

To evaluate the memory and learning parameters, the Morris water maze, IR actophotometer, and rota rod tests were used. Acetylcholinesterase (AChE), lipid peroxidation (LPO), reduced glutathione (GSH), superoxide dismutase (SOD), catalase (CA), and nitrite oxide (NO) assay were among the biochemical parameters that were assessed. This work aims to investigate the effects of these recently synthesized compounds on mice suffering from scopolamine-induced cognitive impairment and to compare those effects with those of donepezil.

## Results

### Behavioral studies

#### Effects of *N*-benzyl-pyridine-2-one derivatives in the MWM test

**Fig. 1:**
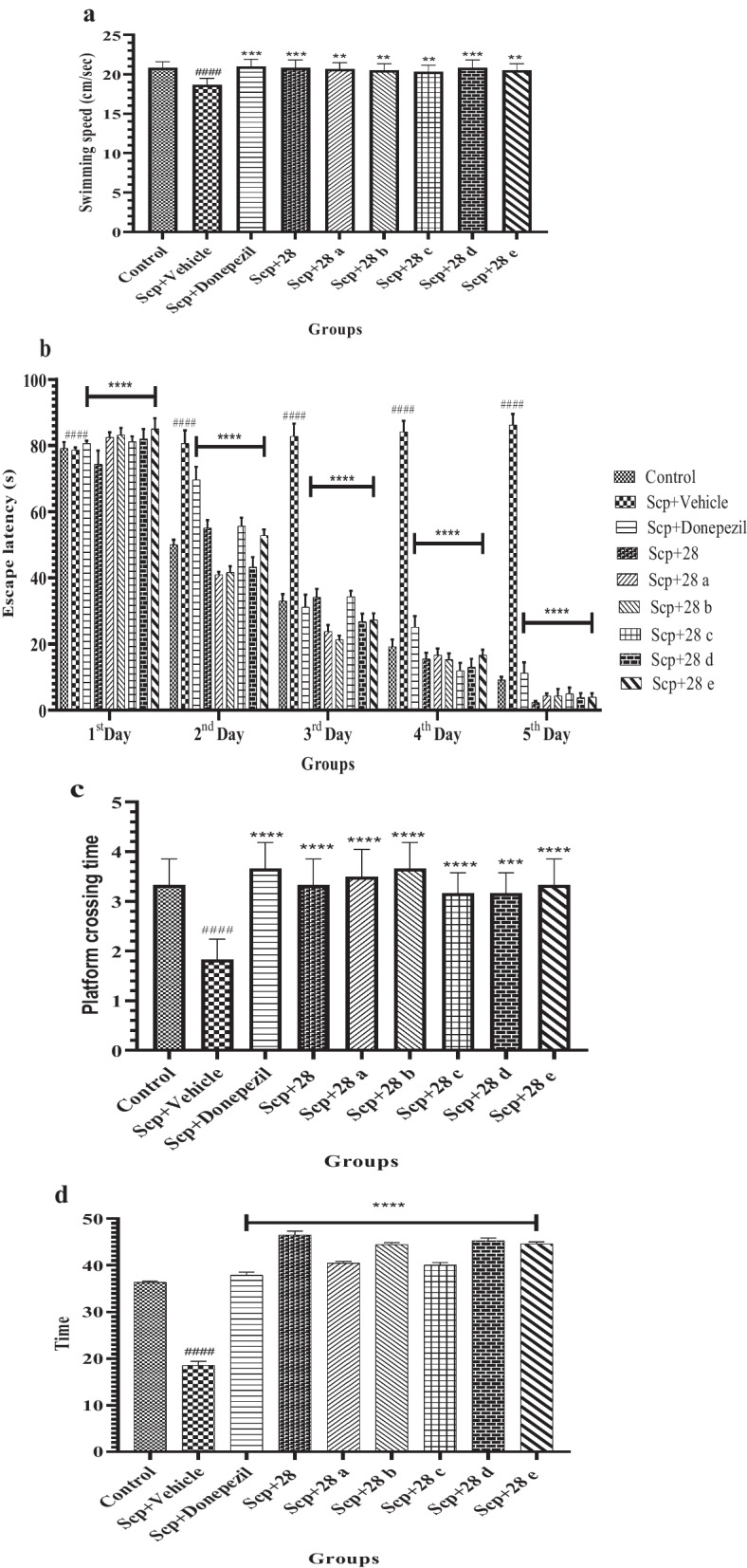
Comparing compounds **28** and **28a**–**e** to the groups of mice given scopolamine, the compounds showed different effects on swim speed (**a**), escape latency (**b**), platform crossing time (**c**), and TSTQ (**d**). Values are presented as Mean ± SD, with n = 6. ^####^p < 0.0001 compares the positive control to the normal control; ****p < 0.0001 compares the treatment groups to the positive control; ***p < 0.001 compares the treatment groups to the positive control; **p < 0.01 compares the treatment groups to the positive control; *p < 0.01 compares the treatment groups to the positive control

The beneficial effects of conventional drug donepezil (2 mg/kg, oral) and novel *N*-benzyl pyridine-2-one derivative compounds (**28** and **28a**–**e**) (2 mg/kg, oral) on scopolamine-induced memory deficits and learning difficulties in mice were examined utilizing the MWM model [12–14]. There was minimum staistical difference in swiiming speed during the MWM test (F (8, 45) = 4.017, p < 0.001). In the behavioral test for memory impairment, mice given scopolamine (1.5 mg/kg, i.p.) showed an increase in escape latency. After receiving treatment with novel *N*-benzyl-pyridine-2-one compounds, escape delay is greatly decreased (F (32, 225) = 148.5, *p* < 0.0001). In the probe trial (day 5) least differences were observed in platform crossing times (F (8, 45) = 7.734, p < 0.0001). However, treatment with these compounds significantly lowers the TSTQ (time spent in target quadrant) in scopolamine-treated mice (F (15, 80) = 777.7, *p* < 0.0001). Compounds **28** and **28d** were found to be the most effective in significantly reducing cognitive deficits when compared to donepezil at 2 mg/kg. The effects of other compounds were equivalent to those of standard medication. (Table 1; Fig. 1a–d).

**Table 1:** Escape latency data and TSQT data of compounds **28**, **28a**–**e**

Compound	Escape latency (s)					TSTQ (s)
	1st day	2nd day	3rd day	4th day	5th day	
Control	79.14 ± 0.19	50 ± 0.15	33 ± 0.21	19.23 ± 0.22	9.15 ± 0.09	36.4 ± 0.22
Scp + vehicle	78.65 ± 0.82	80.64 ± 0.39	82.82 ± 0.37	84.15 ± 0.33	86.25 ± 0.33	18.55 ± 0.88
Scp + donepezil	80.65 ± 0.81	69.66 ± 0.39	31.15 ± 0.37	25.15 ± 0.33	11.25 ± 0.32	37.92 ± 0.68
Scp + **28**	74.33 ± 0.41	55.16 ± 0.27	34.16 ± 0.25	15.5 ± 0.18	2.33 ± 0.47	46.48 ± 0.8
Scp + **28a**	82.53 ± 0.98	41.33 ± 0.89	23.83 ± 0.19	16.66 ± 0.19	4.33 ± 0.74	40.44 ± 0.4
Scp + **28b**	83.16 ± 0.21	41.66 ± 0.18	21.33 ± 0.12	15.33 ± 0.18	4.33 ± 0.25	44.44 ± 0.45
Scp + **28c**	81.16 ± 0.16	55.66 ± 0.25	34.33 ± 0.17	12.0 ± 0.23	5.00 ± 0.18	40.00 ± 0.58
Scp + **28d**	82.00 ± 0.30	43.33 ± 0.29	26.83 ± 0.24	13.0 ± 0.25	3.83 ± 0.13	45.25 ± 0.60
Scp + **28e**	85.0 ± 0.32	52.83 ± 0.18	27.33 ± 0.19	16.66 ± 0.16	4.0 ± 0.11	44.56 ± 0.44

#### Effects of *N*-benzyl-pyridine-2-one derivatives on the locomotor activity

**Fig. 2:**
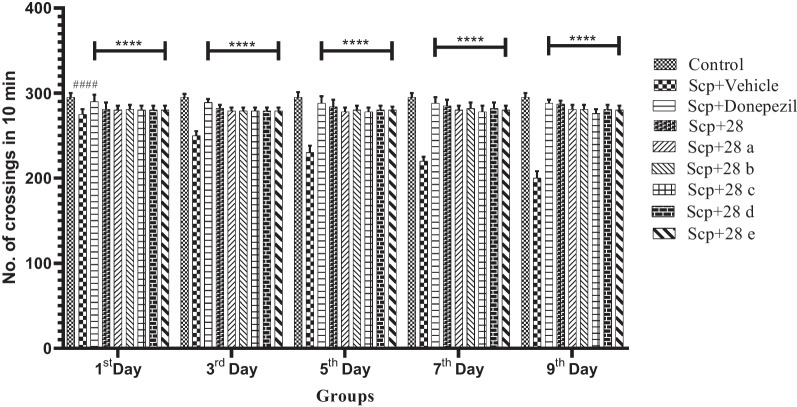
Compounds **28** and **28a**–**e**: their impact on the locomotor activity of mice given scopolamine. The data are presented as Mean ± SD, with n = 6. ####p < 0.0001 compares the positive control to the normal control, and ****p < 0.0001 compares the treatment groups to the positive control

Using an IR-photoactometer, the activity of new *N*-benzyl pyridine-2-one derivatives (**28** and **28a**–**e**) was assessed. Scopolamine treatment led to a significant decrease in locomotor activity when compared to the group that was given only the vehicle. Additionally, administration of these derivatives (2 mg/kg) to mice receiving scopolamine resulted in a noticeably increased locomotor response (F (36, 250) = 16.87, *p* < 0.0001) (Fig. 2).

#### Effects of *N*-benzyl-pyridine-2-one derivatives on the Rotarod activity in scopolamine-induced mice

**Fig. 3:**
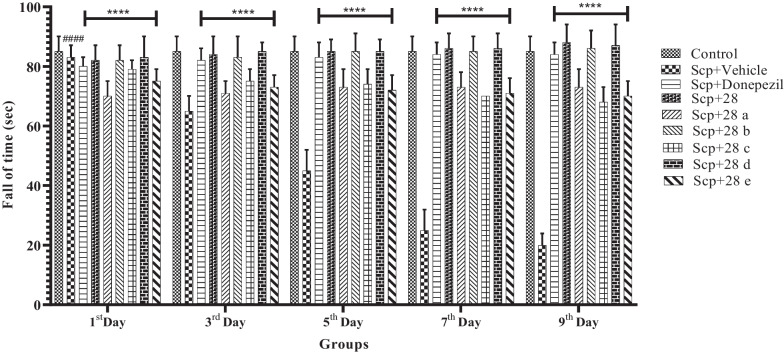
Compounds **28** and **28a**–**e**'s impact on the rotarod performance of mice given scopolamine. The data are presented as Mean ± SD, with n = 6. ####p < 0.0001 compares the positive control to the normal control, and ****p < 0.0001 compares the treatment groups to the positive control

Scopolamine (1.5 mg/kg) medication considerably decreased muscle grip strength, according to the results of the rotarod test. Additionally, mice given *N*-benzyl pyridine-2-one (2 mg/kg, p.o.) after receiving scopolamine treatment demonstrated a significant increase in the strength of their muscles' grip (F (8, 36) = 9.059, *p* < 0.0001) (Fig. 3).

### Biochemical tests

#### Effect of *N*-benzyl-pyridine-2-one derivatives on AChE activity

**Fig. 4:**
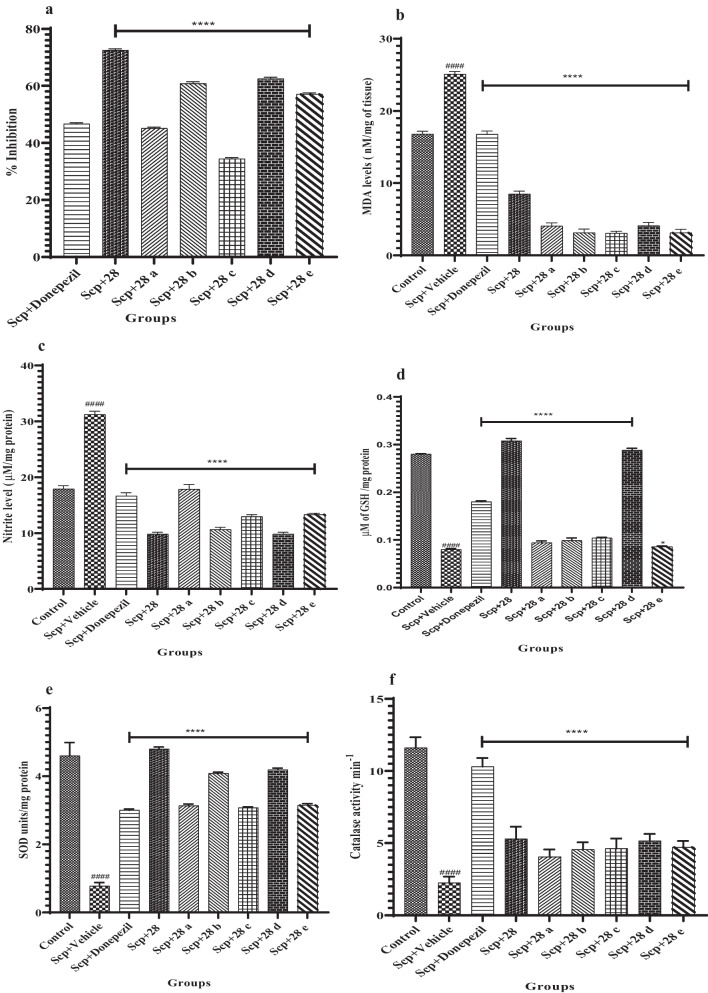
The impact of compounds **28** and **28a**–**e** on the following parameters was measured in comparison to the mice treated with scopolamine: acetylcholinesterase level (**a**), malondialdehyde (MDA) level, a measure of lipid peroxidation (**b**), nitrite level (**c**), GSH levels (**d**), SOD activity (**e**), catalase level, and an antioxidant profile measure (**f**). The values are presented as Mean ± SD, n = 6 ####p < 0.0001 (positive control is contrasted with the normal control), ****p < 0.0001, respectively

When scopolamine is administered, brain AChE levels rise in comparison to the control group. However, donepezil treatment resulted in *N*-benzyl pyridine-2-one derivatives **28** and **28a**–**e**, which significantly inhibit the amount of AChE in the brain when compared to the comparable scopolamine-treated groups (F (6, 35) = 4407, *p* < 0.0001) (Table 1; Fig. 4a).

#### Effect of *N*-benzyl-pyridine-2-one derivatives on brain LPO, NO, GSH, SOD and CA levels in scoplamine-treated mice

When compared to a vehicle-treated mouse brain, the administration of scopolamine markedly increased LPO and NO levels and decreased GSH, SOD, and CA activity. In contrast, scopolamine-treated mice showed increased GSH (F (8, 45) = 4987, p < 0.0001), SOD (F (8, 45) = 450.9, p < 0.0001), and CA (F (8, 45) = 156.3, p < 0.0001) activities, while NO and LPO levels (F (8, 45) = 2235, p < 0.0001) and NO levels (F (8, 45) = 1016, p < 0.0001) were decreased in the latter group (Fig. 4b–f). Of all the substances, treatment with compounds **28** and **28d** was the most successful in returning the biochemical mediators to their most normal state and significantly reduced the reactive oxygen and nitrogen species linked to the neuroinflammation mechanism (Table 1).

## Discussion

**Fig. 5:**
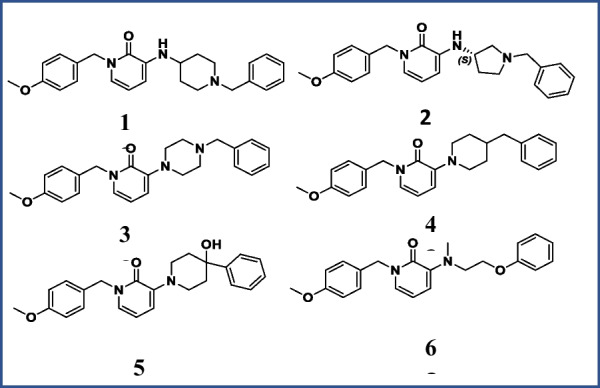
Structures of novel *N*-benzyl-pyridine-2-one derivatives (**28**, **28a**–**e**, 2 mg/kg)

In this study, we looked into how new *N*-benzyl pyridine-2-one compounds affected the memory and learning deficits in mice. Since scopolamine has been shown to impair memory by blocking muscarinic cholinergic receptors in the brain, we used it to induce cognitive impairments in mice [15]. We evaluated the effects of these novel derivatives on spatial learning, memory, and locomotor activity using the MWM, rotarod, and IR actophotometer tests (Fig. 5).

In our studies, a dose of 2 mg/kg was chosen based on reports from the past literature [16]. The amelioration of scopolamine-induced memory deficits by *N*-benzyl-pyridine-2-one derivatives raises the possibility that these derivatives could be beneficial in treating cholinergic blockade-induced cognitive dysfunction. To completely comprehend the mechanisms underlying the neurotransmitter system(s) involvement in cognitive function, more research is necessary (Table 2).

**Table 2:** Biochemical estimation data of compounds **28**, **28a**–**e**

Compound	% Inhibition AChE	LPO assay, MDA levels (Nm/mg of tissue)	Nitrite assay, nitrite conc. (Μm/mg) protein	GSH assay, μmol of GSH/mg Protein	SOD assay, SOD units/mg protein	Catalase assay, catalase activity min^−1^
Control	–	16.58 ± 0.04	17.88 ± 0.05	0.02 ± 0.001	4.60 ± 0.03	11.66 ± 0.05
Scp + vehicle	–	25.09 ± 0.03	31.21 ± 0.04	0.08 ± 0.002	0.78 ± 0.01	2.25 ± 0.04
Scp + donepezil	46.6 ± 0.04	16.76 ± 0.04	16.63 ± 0.05	0.18 ± 0.002	3 ± 0.03	10.3 ± 0.05
Scp + **28**	72.5 ± 0.04	8.50 ± 0.04	9.80 ± 0.03	0.3 ± 0.005	4.8 ± 0.04	5.28 ± 0.03
Scp + **28a**	45 ± 0.04	4.08 ± 0.04	17.82 ± 0.08	0.09 ± 0.003	3.13 ± 0.05	4.05 ± 0.05
Scp + **28b**	60.8 ± 0.05	3.13 ± 0.05	10.64 ± 0.04	0.09 ± 0.005	4.08 ± 0.04	4.56 ± 0.04
Scp + **28c**	34.3 ± 0.04	3.07 ± 0.02	12.96 ± 0.04	0.1 ± 0.001	3.07 ± 0.02	4.62 ± 0.05
Scp + **28d**	62.5 ± 0.05	4.09 ± 0.04	9.80 ± 0.03	0.2 ± 0.004	4.19 ± 0.04	5.14 ± 0.05
Scp + **28e**	57 ± 0.03	3.14 ± 0.04	13.32 ± 0.02	0.08 ± 0.001	3.14 ± 0.04	4.71 ± 0.04

Scopolamine has been linked to oxidative stress in the brain and cholinergic neurotransmitter interference in the past [17, 18]. These processes are both involved in the pathogeneses of AD. Antioxidants are used as one therapy against neurodegenerative diseases because oxidative stress can damage brain cells and other neural tissue, speed up aging, and cause premature cell apoptosis [19].

Learning and memory may be impacted by oxidative stress-induced brain damage [20]. In the same context, a number of researchers discovered that lower antioxidant reserves and higher brain LPO and NO concentrations were associated with scopolamine-induced amnesia and memory impairment in rats [21–23]. In the current work, this was illustrated by scopolamine-induced increases in brain LPO and NO levels along with decreased levels of GSH, SOD, and CA activity. On the other hand, *N*-benzyl-pyridine-2-one derivatives lead to decreased levels of brain lipid peroxidation and nitrite and increased levels of GSH, SOD, and CA activity.

To summarize, the outcomes of this pre-clinical investigation aligned with the findings of the in-silico evaluations noted in the prior research, suggesting that these synthetic *N*-benzyl-pyridine-2-one derivatives could be helpful in the creation of novel and effective neuro-pharmacological medication candidates. The mechanism or mechanisms of action of the experimental findings, however, require further investigation.

## Conclusions

To sum up, new *N*-benzyl-pyridine-2-one derivatives have neuroprotective effects against mice's scopolamine-induced cognitive decline. All of the synthesized compounds, including **28** and **28a**–**e**, significantly reduced scopolamine's capacity to induce AChE activity, oxidative stress, and nitrosative stress in the MWM animal model. In the rota rod test, they also improved muscle grip strength and locomotor activity. The *N*-benzyl-pyridine-2-one derivatives' compounds **28** and **28d** showed the greatest efficacy and provided better neuroprotection than donepezil, the industry standard. The study suggests that these novel derivatives of *N*-benzyl-pyridine-2-one may be useful in treating diseases like Alzheimer's that cause cognitive impairments.

## Methods

### Chemicals

Scopolamine hydrobromide and Donepezil hydrochloride were brought from Merck (Merck KGaA, Darmstadt, Germany).Trichloroacetic acid, acetylthiocholine iodide, 5,5′-dithiobisnitrobenzoic acid (DTNB), hydroxylamine hydrochloride, and ferric chloride, were brought from Sigma-Aldrich (St. Louis, MO, USA). Synthetic pyridine-2-one derivatives **28**, **28a**–**e** previously synthesized by our group were used in the study [11]. All other reagents and chemicals utilized in the investigation were of analytical grade.

### Equipments

Actophotometer (IMCORP, Ambala, India), Rotarod apparatus (Techno, Ambala, India), Centrifuge (Remi, India), and UV–visible spectrophotometer (Shimadzu, UV1800, Japan) were used in this study.

### Animals

In this study, male Swiss albino mice weighing 25–30 g and aged 7–9 weeks were employed. The purchase of these animals came from LLRUVAS in Hisar, Haryana. The Institutional Animal Ethics Committee (IAEC) granted prior authorization for the experimental procedures, and the protocols were approved with protocol number BV/IAEC/4278/2021.

The mice were housed in groups of six in a standard laboratory setting with a 12-h light/dark cycle, free access to food and drink, and a temperature of 25 ± 2 °C and 60 ± 2% relative humidity. The regulations of the Committee for the Purpose of Control and Supervision of Animal Experiments on Animals (CPCSEA) were adhered to in the housing, care, and handling of the animals.

### Dose administration

**Fig. 6:**
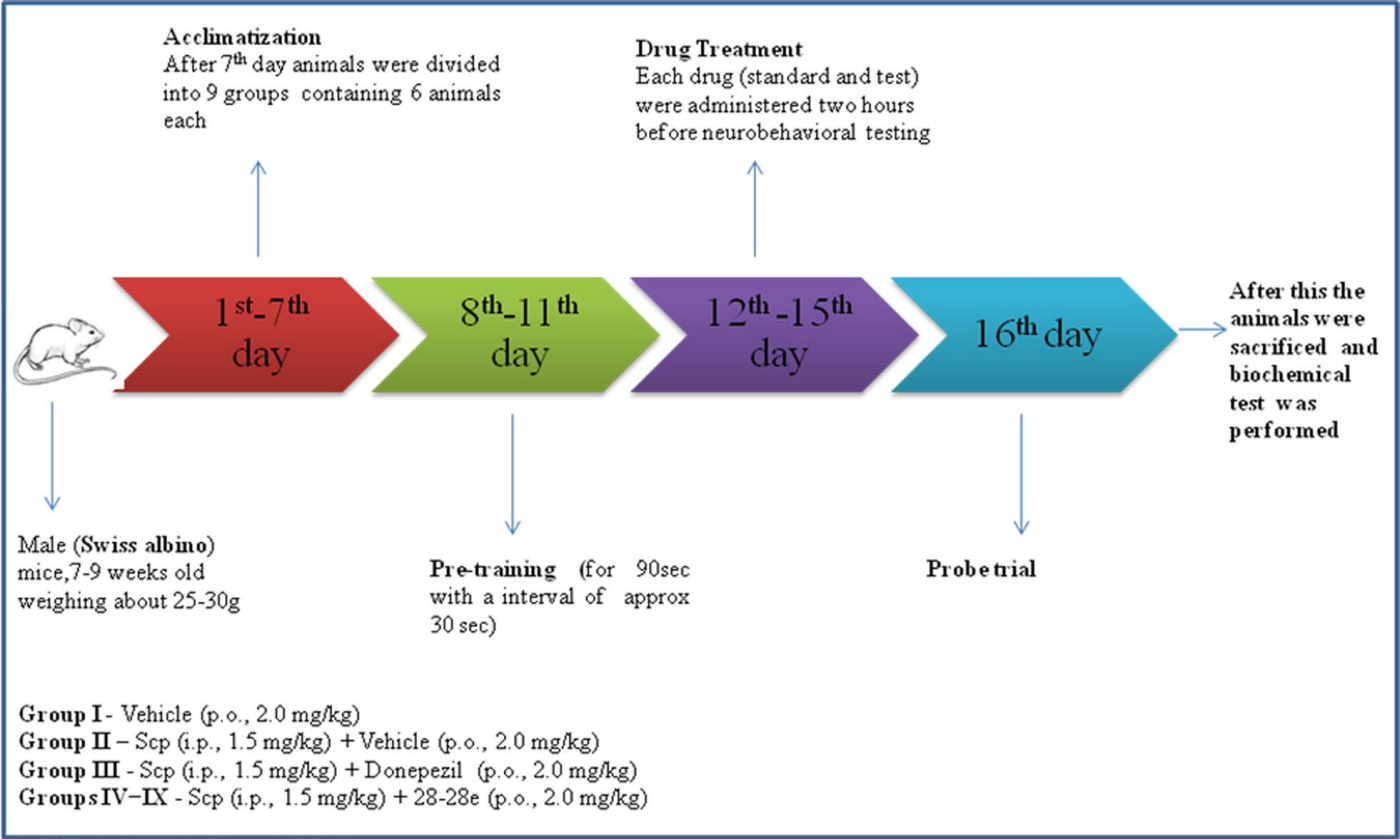
Animal experimentation scheme

After a suitable period of acclimation, the animals were divided into nine groups (I-IX), each containing six animals. For five days, at least two hours prior to evaluation, scopolamine (i.p.) was administered. It was also administered in combination with donepezil (peroral (p.o.)) and synthetic compounds (p.o.). Groups 1 and 2 (vehicle treated), 3 (scopolamine combined with donepezil, p.o., 2.0 mg/kg), and 4–9 (scopolamine combined with synthetic compounds [**28**, **28a**–**e**, p.o., 2.0 mg/kg]) were the other groups. The animals were allowed to acclimate to the lab setting for seven days prior to any experiments. Every experiment was conducted every day from 8:00 a.m. to 6:00 p.m. (Fig. 6).

### Behavioral tests

#### Morris water maze (MWM)

Animals were continuously assessed using the MWM model for five days, which included training and probe trials. It is made up of a circular water tank that is 120 cm in diameter and 60 cm tall, with a black painted interior, and is filled with water (25 ± 2 ˚C) up to a height of 55 cm. The tank was set up in a dimly lit laboratory, and it was divided into an equal quadrant of four (P1–P4) using wires that were anchored at the edge of the pool at an angle to one another. At the pool's designated area (P4), a stage with an upper face measuring 6 by 6 cm^2^ was positioned 1 cm below the surface. Throughout the assessment, the stage's location remained constant. For four days in a row, the animals received daily training trials that lasted 90 s, with a 30-s break in between trials. Throughout the examination, the animals were exposed to water pools by moving their heads towards the wall from any of the four quadrants (P1–P4) and being given ninety seconds to identify the stage placed in a pool. If the animals couldn't reach the stage in ninety seconds, they were gently guided onto it under supervision. One indicator of whether cognitive deficits have improved is the time it took the mice on the fifth day to travel from the beginning point to the hidden stage in the desired portion (escape latency) [24].

#### Locomotor activity

The locomotor activity was monitored with an actophotometer. An electric impulse generated by each beam interruption was shown on the x- or y-axis by a digital counter. The apparatus was placed in a testing room with sound and light attenuation, ventilation, and darkness. Ten minutes were spent observing each animal's activity after it was placed in the exercise cage. Both before and after the drug delivery, the test is conducted. To ascertain whether locomotor activity has increased or decreased, one uses the photocell count [25].

#### Rotarod activity

The rotarod was utilized to gauge each animal's ability to grip. Before beginning treatment, each mouse was trained to become accustomed to the rotarod apparatus (Orchid Scientific, India). The animal was fixed to a 7 cm-diameter spinning rod. (25 rpm in speed). Over the course of five minutes, each mouse received three trials, with the experiment's end time of 180 being maintained. A fall in time was observed as the typical outcome [26].

#### Dissection and homogenization

The mice were decapitated while under mild anesthesia as a means of sacrificing them following the last day of behavioral testing. The brains were carefully removed to create brain tissue homogenates as soon as they were beheaded. Using a homogenizer and cold 0.1 M phosphate buffer, tissue homogenates (TSH) (10% w/v) were created. 7.4 pH adjusted. The supernatant from homogenates was centrifuged at 12,000 rpm for 20 min at 4 °C in order to test biological parameters.

### Biochemical tests

#### Acetylcholinesterase (AChE) estimation

The concentration of the cholinergic marker AChE throughout the whole brain was estimated using the Ellman method. Chemically speaking, DTNB is another name for Ellman's reagent. This homogenate was mixed with 2.7 mL of phosphate buffer, 0.1 mL of DTNB, and 5 min of standing. After adding 0.1 mL of freshly prepared acetylthiocholine iodide (pH 8), 412 nm was used to measure the absorbance [27, 28].

#### Estimation of lipid peroxidation (LPO)

The malondialdehyde level, a measure of LPO, was computed using Wills' description of the chemical reactions to thiobarbituric acid [29]. After mixing 0.5 mL of TSH and 0.5 mL of Tris–HCl, the mixture was exposed to 1.0 mL of 10% trichloroacetic acid for two hours at 37 °C. The mixture was centrifuged once more after being incubated. The sample tubes were filled with 0.67% thiobarbituric acid and supernatant, and then heated for ten minutes. To measure the absorbance at 532 nm, 1.0 mL of double-distilled water was added after chilling. Using the extinction coefficient of a chromophore, the reactive chemicals were calculated and presented as moles of malondialdehyde/mg of protein (1.56 10^5^ M^−1^ cm^−1^).

#### Reduced glutathione level (GSH)

The process described by Jollow et al. was followed. One milliliter (1.0 mL) of 4% sulfosalicylic acid was used to induce the TSH (1 mL). The product was stored at 4 °C for an hour after being centrifuged for 10 min at 1200 rpm. The analyte contained the supernatant, phosphate buffer, and Ellman's reagent in its 3.0 mL volume. At a wavelength of 412 nm, the absorbance of the yellow solution was promptly measured, and the results were expressed as nmol of GSH/mg of protein [30].

#### Superoxide dismutase activity (SOD)

The analyses were made using 50 mM sodium carbonate, 0.1 mM EDTA, and 96 mM nitroblue tetrazolium (NBT) using Kono's method. The analytes TSH (0.05 mL), hydroxylamine, and other are contained in the sample tubes in 2.0 mL each. The mixture was monitored for absorbance changes every 30 or 60 s for two minutes at 560 nm [31].

#### Catalase (CA)

The activity was evaluated using the Luck-reported evaluation method. The analytes used to calculate the optical density were hydrogen peroxide (0.019 M), TSH (1.0 mL, 0.019 M), and phosphate buffer (1.95 mL, 0.05 M, pH 7.0). (five milliliters). The results were expressed as M hydrogen peroxide decomposition/min/mg protein [32].

#### Nitrite oxide (NO)

Nitric oxide levels in TSH were determined using Greiss' reagent, which is a solution of 0.5% sulfonamide, 2.5% phosphoric acid, and 0.05% naphthyl ethylenediamine. Greiss reagent absorbance and TSH were measured at 540 nm, and the sodium nitrite standard curve was used to calculate the amount of nitrite [33].

### Statistical analysis

The results are shown as mean ± SD. The results were analyzed by one- and two-way analysis of variance (ANOVA) followed by Tukey’s test. Statistical analysis was carried out using Graph Pad Prism 8.3.0. The cutoff for statistical significance was p < 0.05.

### Supplementary Information

**Additional file 1:** Graphical abstract.** Figure S1.** Comparing compounds 28 and 28a-e to the groups of mice given scopolamine, the compounds showed different effects on swim speed (**a**), escape latency (**b**), platform crossing time (**c**), and TSTQ (**d**). Values are presented as Mean ± SD, with n = 6. ####p < 0.0001 compares the positive control to the normal control; ****p < 0.0001 compares the treatment groups to the positive control; ***p < 0.001 compares the treatment groups to the positive control; **p < 0.01 compares the treatment groups to the positive control; *p < 0.01 compares the treatment groups to the positive control. **Figure S2.** Compounds 28 and 28a-e: their impact on the locomotor activity of mice given scopolamine. The data are presented as Mean ± SD, with n = 6. ####p < 0.0001 compares the positive control to the normal control, and ****p < 0.0001 compares the treatment groups to the positive control. **Figure S3**. Compounds 28 and 28a-e's impact on the rotarod performance of mice given scopolamine. The data are presented as Mean ± SD, with n = 6. ####p < 0.0001 compares the positive control to the normal control, and ****p < 0.0001 compares the treatment groups to the positive control. **Figure S4.** Compounds 28 and 28a-e's impact on the rotarod performance of mice given scopolamine. The data are presented as Mean ± SD, with n = 6. ####p < 0.0001 compares the positive control to the normal control, and ****p < 0.0001 compares the treatment groups to the positive control. **Figure S5.** Structures of  novel N-benzyl-pyridine-2-one derivatives (28, 28a-e, 2 mg/kg).

## Data Availability

All data generated during this study are included in this published article and its suplementary information files.

## Abbreviations

Ach: Acetylcholine
AChE: Acetylcholinesterase
AD: Alzheimer’s disease
ANOVA: Analysis of variance
BChE: Butyrylcholinesterase
CA: Catalase
ChAT: Choline acetyltransferase
CPCSEA: Committee for the Purpose of Control and Supervision of Experiments on Animals
DTNB: 5,5′-Dithio-bis-(2-nitrobenzoic acid)
EDTA: Ethylenediaminetetraacetic acid
FDA: Food and Drug Administration
IAEC: Institutional Animal Ethics Committee
LPO: Lipid peroxidation
MWM: Morris water maze
NO: Nitric oxide level
NBT: Nitroblue tetrazolium
SOD: Superoxide dismutase activity
TSH: Tissue homogenates
TSTQ: Time spent in target quadrant

## References

[1] Zhang J, Shi L, Shen Y (2022). The retina: a window in which to view the pathogenesis of Alzheimer’s disease. Ageing Res Rev.

[2] Lynch C (2020). World Alzheimer Report 2019: attitudes to demnetia, a global survey. Alzheimer’s Dement.

[3] Pant S, Gupta M, Anthwal T, Chauhan M, Nain S (2023). Neuroprotective effects of novel pyrrolidine-2-one derivatives on scopolamine-induced cognitive impairment in mice: Behavioral and biochemical analysis. Pharmacol Biochem Behav.

[4] Bhatia V, Sharma S (2021). Role of mitochondrial dysfunction, oxidative stress and autophagy in progression of Alzheimer’s disease. J Neurol Sci.

[5] Talesa VN (2001). Acetylcholinesterase in Alzheimer’s disease. Mech Ageing Dev.

[6] Arce MP, Rodríguez-Franco MI, González-Muñoz GC, Pérez C, López B, Villarroya M (2009). Neuroprotective and cholinergic properties of multifunctional glutamic acid derivatives for the treatment of Alzheimer’s disease. J Med Chem.

[7] Adlimoghaddam A, Neuendorff M, Roy B, Albensi BC (2018). A review of clinical treatment considerations of donepezil in severe Alzheimer’s disease. CNS Neurosci Ther.

[8] Marucci G, Buccioni M, Dal Ben DD, Lambertucci C, Volpini R, Amenta F (2021). Efficacy of acetylcholinesterase inhibitors in Alzheimer’s disease. Neuropharmacology.

[9] Ayaz M, Ullah F, Sadiq A, Kim MO, Ali T (2019). Editorial: Natural products-based drugs: potential therapeutics against Alzheimer’s disease and other neurological disorders. Front Pharmacol.

[10] Ahmed S, Khan ST, Zargaham MK, Khan AU, Khan S, Hussain A (2021). Potential therapeutic natural products against Alzheimer’s disease with reference of Acetylcholinesterase. Biomed Pharmacother.

[11] Gupta M, Kumar A, Prasun C, Nair MS, Kini SG, Yadav D (2023). Design, synthesis, extra-precision docking, and molecular dynamics simulation studies of pyrrolidin-2-one derivatives as potential acetylcholinesterase inhibitors. J Biomol Struct Dyn.

[12] Kumar P, Kumar A (2009). Protective effect of rivastigmine against 3-nitropropionic acid-induced Huntington's disease like symptoms: possible behavioural, biochemical and cellular alterations. Eur J Pharmacol.

[13] Goverdhan P, Sravanthi A, Mamatha T (2012). Neuroprotective effects of meloxicam and selegiline in scopolamine-induced cognitive impairment and oxidative stress. Int J Alzheimers Dis.

[14] Saxena G, Singh SP, Agrawal R, Nath C (2008). Effect of donepezil and tacrine on oxidative stress in intracerebral streptozotocin-induced model of dementia in mice. Eur J Pharmacol.

[15] Giovannini MG, Spignoli G, Carlà V, Pepeu G (1991). A decrease in brain catecholamines prevents oxiracetam antagonism of the effects of scopolamine on memory and brain acetylcholine. Pharmacol Res.

[16] Gupta M, Ojha M, Yadav D, Pant S, Yadav R (2020). Novel benzylated (pyrrolidin-2-one)/(imidazolidin-2-one) derivatives as potential anti-Alzheimer's agents: synthesis and pharmacological investigations. ACS Chem Neurosci.

[17] Lee JS, Kim HG, Han JM, Kim DW, Yi MH, Son SW (2014). Ethanol extract of Astragali Radix and Salviae Miltiorrhizae Radix, Myelophil, exerts anti-amnesic effect in a mouse model of scopolamine-induced memory deficits. J Ethnopharmacol.

[18] Shi J, Liu Q, Wang Y, Luo G (2010). Coadministration of huperzine A and ligustrazine phosphate effectively reverses scopolamine-induced amnesia in rats. Pharmacol Biochem Behav.

[19] Valko M, Leibfritz D, Moncol J, Cronin MT, Mazur M, Telser J (2007). Free radicals and antioxidants in normal physiological functions and human disease. Int J Biochem Cell Biol.

[20] Fukui K, Omoi NO, Hayasaka T, Shinnkai T, Suzuki S, Abe K (2002). Cognitive impairment of rats caused by oxidative stress and aging, and its prevention by vitamin E. Ann N Y Acad Sci.

[21] Khalifa AE (2004). Pro-oxidant activity of zuclopenthixol in vivo: differential effect of the drug on brain oxidative status of scopolamine-treated rats. Hum Exp Toxicol.

[22] Fan Y, Hu J, Li J, Yang Z, Xin X, Wang J (2005). Effect of acidic oligosaccharide sugar chain on scopolamine-induced memory impairment in rats and its related mechanisms. Neurosci Lett.

[23] Jeong EJ, Ma CJ, Lee KY, Kim SH, Sung SH, Kim YC (2009). KD-501, a standardized extract of Scrophularia buergeriana has both cognitive-enhancing and antioxidant activities in mice given scopolamine. J Ethnopharmacol.

[24] Morris R (1984). Developments of a water-maze procedure for studying spatial learning in the rat. J Neurosci Methods.

[25] Vogel HG, Schlkens BA, Sandow J (2002). Drug effects on learning and memory. Drug Discov Eval Pharmacol Assays.

[26] Kulkarni SK. Handbook of experimental pharmacology. Vallabh Prakashan; 1987.

[27] Abhinav K, Jogender M, Madhusudana K, Naidu VGM, Gupta YK (2010). Anti-amnesic activity of Vitex negundo in scopolamine induced amnesia in rats. Pharmacol Pharm.

[28] Kumar A, Dogra S, Prakash A (2009). Neuroprotective effects of *Centella asiatica* against intracerebroventricular colchicine-induced cognitive impairment and oxidative stress. Int J Alzheimers Dis.

[29] Wills ED (1966). Mechanisms of lipid peroxide formation in animal tissues. Biochem J.

[30] Ellman GL (1959). Tissue sulfhydryl groups. Arch Biochem Biophys.

[31] Kono Y (1978). Generation of superoxide radical during autoxidation of hydroxylamine and an assay for superoxide dismutase. Arch Biochem Biophys.

[32] Luck H, Bergmeyer HU (1965). Catalase. Method of enzymatic analysis.

[33] Green LC, Wagner DA, Glogowski J, Skipper PL, Wishnok JS, Tannebaum SR (1982). Analysis of nitrate, nitrite, and [15N] nitrate in biological fluids. Anal Biochem.

