# Supplementary material for: The memory ameliorating effects of novel N-benzyl pyridine-2-one derivatives on scopolamine-induced cognitive deficits in mice
Source: Lab Anim Res. 2024 Mar 11;40:9. doi: 10.1186/s42826-023-00187-y (PMC10926586; doi:10.1186/s42826-023-00187-y)
Supplement: Supplementary file 1 — Additional file 1: Graphical abstract. Figure S1. Comparing compounds 28 and 28a-e to the groups of mice given scopolamine, the compounds showed different effects on swim speed (a), escape latency (b), platform crossing time (c), and TSTQ (d). Values are presented as Mean ± SD, with n = 6. ####p < 0.0001 compares the positive control to the normal control; ****p < 0.0001 compares the treatment groups to the positive control; ***p < 0.001 compares the treatment groups to the positive control; **p < 0.01 compares the treatment groups to the positive control; *p < 0.01 compares the treatment groups to the positive control. Figure S2. Compounds 28 and 28a-e: their impact on the locomotor activity of mice given scopolamine. The data are presented as Mean ± SD, with n = 6. ####p < 0.0001 compares the positive control to the normal control, and ****p < 0.0001 compares the treatment groups to the positive control. Figure S3. Compounds 28 and 28a-e's impact on the rotarod performance of mice given scopolamine. The data are presented as Mean ± SD, with n = 6. ####p < 0.0001 compares the positive control to the normal control, and ****p < 0.0001 compares the treatment groups to the positive control. Figure S4. Compounds 28 and 28a-e's impact on the rotarod performance of mice given scopolamine. The data are presented as Mean ± SD, with n = 6. ####p < 0.0001 compares the positive control to the normal control, and ****p < 0.0001 compares the treatment groups to the positive control. Figure S5. Structures of novel N-benzyl-pyridine-2-one derivatives (28, 28a-e, 2 mg/kg). [file 42826_2023_187_MOESM1_ESM.doc]

**Supplementary information**

**The memory ameliorating effects of novel N-benzyl pyridine-2-one derivatives on scopolamine-induced cognitive deficits in mice**

Swati Pant, Mohan Gupta, Tulika Anthwal, Monika Chauhan and Sumitra Nain*

*Department of Pharmacy, Banasthali Vidyapith, Banasthali, Rajasthan, India*

Corresponding author: Sumitra Nain, Department of Pharmacy, Banasthali Vidyapith, Banasthali, Tonk, Rajasthan, India; Tel: +91-7665555900; E-mails: [nainsumitra@gmail.com](mailto:nainsumitra@gmail.com)

**Graphical Abstract**

**Fig. 1** . Comparing compounds 28 and 28a-e to the groups of mice given scopolamine, the compounds showed different effects on swim speed (a), escape latency (b), platform crossing time (c), and TSTQ (d). Values are presented as Mean±SD, with n = 6. ####p<0.0001 compares the positive control to the normal control; ****p<0.0001 compares the treatment groups to the positive control; ***p<0.001 compares the treatment groups to the positive control; **p<0.01 compares the treatment groups to the positive control; *p<0.01 compares the treatment groups to the positive control.

**Fig. 2** Compounds 28 and 28a-e: their impact on the locomotor activity of mice given scopolamine. The data are presented as Mean±SD, with n = 6. ####p<0.0001 compares the positive control to the normal control, and ****p<0.0001 compares the treatment groups to the positive control.

**Fig. 3** Compounds 28 and 28a-e's impact on the rotarod performance of mice given scopolamine. The data are presented as Mean±SD, with n = 6. ####p<0.0001 compares the positive control to the normal control, and ****p<0.0001 compares the treatment groups to the positive control.

**Fig. 4**. The impact of compounds 28 and 28a-e on the following parameters was measured in comparison to the mice treated with scopolamine: acetylcholinesterase level (a), malondialdehyde (MDA) level, a measure of lipid peroxidation (b), nitrite level (c), GSH levels (d), SOD activity (e), catalase level (f). The values are presented as Mean±SD, n=6 ####p<0.0001 (positive control is contrasted with the normal control), ****p<0.0001, respectively.

**Fig. 5** . Structures of novel N-benzyl-pyridine-2-one derivatives (28, 28a-e, 2 mg/kg).

**18**

**28a**

**38b**

**428c**

**58d**

**68e**
